# A sustainable resistive switching memory device based on organic keratin extracted from hair

**DOI:** 10.1039/c8ra10643f

**Published:** 2019-04-23

**Authors:** Bolin Guo, Bai Sun, Wentao Hou, Yuanzheng Chen, Shouhui Zhu, Suangsuo Mao, Liang Zheng, Ming Lei, Bing Li, Guoqiang Fu

**Affiliations:** School of Physical Science and Technology, Key Laboratory of Advanced Technologies of Materials, Ministry of Education of China, Southwest Jiaotong University Chengdu 610031 China bsun@swjtu.edu.cn; Department of Electromechanical Measuring and Controlling, School of Mechanical Engineering, Southwest Jiaotong University Chengdu Sichuan 610031 China fuguoqiang@swjtu.edu.cn; Key Laboratory of Magnetic Levitation Technologies and Maglev Trains, Ministry of Education of China, Superconductivity and New Energy R&D Center (SNERDC), Southwest Jiaotong University Chengdu Sichuan 610031 China; College of Materials Science and Technology, Nanjing University of Aeronautics and Astronautics (NUAA) Yudao Street 29 210016 Nanjing China; School of Electrical Engineering Southwest Jiaotong University Chengdu Sichuan 610031 China

## Abstract

It is the consensus of researchers that the reuse of natural resources is an effective way to solve the problems of environmental pollution, waste and overcapacity. Moreover, compared with the case of inorganic materials, the renewability of natural biomaterials has great prominent advantages. In this study, keratin, which was first extracted from hair due to its high content in hair, was chosen as a functional layer for the fabrication of a resistance switching device with the Ag/keratin/ITO structure; in this device, a stable resistive switching memory behavior with good retention characteristic was observed. *Via* mechanism analysis, it is expected that there is hopping conduction at low biases, and the formation of a conductive filament occurs at high biases. Furthermore, our device exhibited a stable switching behavior with different conductive materials (Ti and FTO) as bottom electrodes, and the influence of Ag and graphite conductive nanoparticles (NPs) doped into the keratin layer on the switching performance of the device was also investigated. This study not only suggests that keratin is a potential biomaterial for the preparation of memory devices, but also provides a promising route for the fabrication of bio-electronic devices with non-toxicity, degradability, sustainability *etc.*

## Abstract 2

This study suggests that keratin is a potential biomaterial for the preparation of memory devices with non-toxicity, degradability and sustainability.

## Graphical abstract

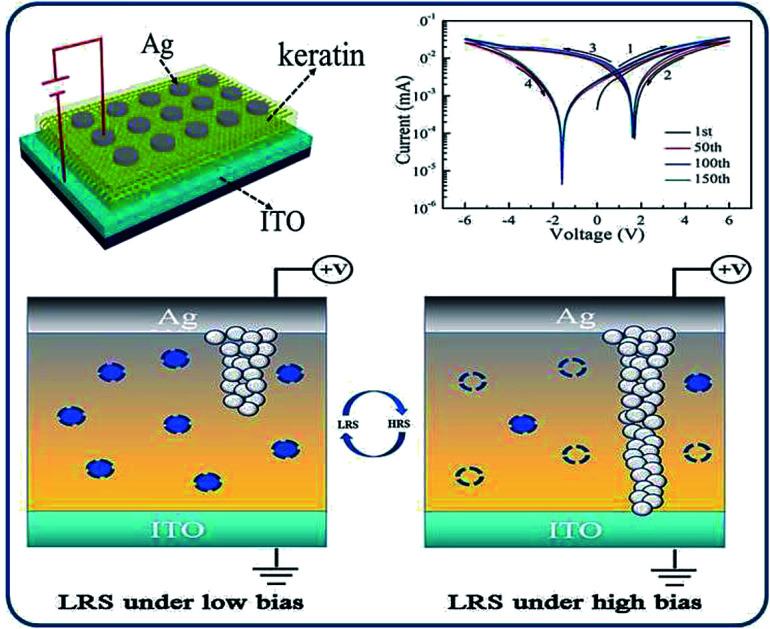

## 1. Introduction

Currently, as is well-known, natural biomaterials, which have excellent biocompatibility, biodegradability, sustainability, environmental friendliness and abundant resources, have attracted extensive attention in the preparation of resistive switching memory devices;^1–7^ moreover, a biomaterial-based electronic device can overcome some issues, such as shortage of resources, biological incompatibility and toxicity, that have been found in inorganic materials.^5–9^ In recent studies, a variety of natural biomaterials, including leaves, DNA, sericin, fibroin and lignin, have shown superior resistive switching properties.^10–14^ Most of these materials have large HRS/LRS resistance ratio and good retention characteristic, which make them hold the potential to be applied in bio-electronic devices.^15–17^ Moreover, since bio-electronic devices have low energy consumption and are pollution-free, they have attracted significant attention. In particular, this kind of green electronic device is more in line with the environmental protection concept of modern society.

Keratin, which is a renewable protein, is widely present in the hair and epithelial tissues of animals, where it is usually present in high amounts: for example, the content of keratin can reach 90% in human hair.^18,19^ In addition, as a kind of scleroprotein, its chemical and physical properties are stable: it cannot be absorbed by the body, it is non-toxic, and it is non-polluting and resistant to acid and alkali.^20^ Therefore, keratin can be used as an excellent dielectric material for the preparation of electronic devices that can be implanted into the human body in the future.^21^ Nowadays, a number of studies have been reported on protein-based resistive switching memory devices,^15,17^ for which the most commonly studied proteins are silk fibroin and sericin;^11,12^ however, although keratin, as the most abundant protein, can be easily obtained and produced on a large scale, to date, it has not been investigated with respect to the abovementioned application. Moreover, the emergence of some protein-based bio-electronic devices, such as transistors, diodes and optical waveguides, has led to significant application prospects of keratin.^22–26^ In addition, the application of keratin in solid-state bio-electronic devices is a good example of recycling of waste resources.

Indeed, resistive random access memory (RRAM) is most promising for next-generation high-performance memory devices, which are expected to have fast memory speed, high storage density, low cost, non-volatility and good cycling endurance;^10,13^ thus, they can overcome the shortcomings, such as high cost and power consumption during a refresh cycle, of some existing memory devices such as dynamic random access memory (DRAM) devices. Flash memory devices, which need a fair amount of time for erasing and rewriting data, and hard-disk drives (HDD) are slow to access and easily damaged.^27^ Traditionally, the resistive switching devices can be classified into volatile and non-volatile switching devices.^17,28^ Among them, the volatile switching devices are mainly used in dynamic random access memory (DRAM) and static random access memory (SRAM) cells,^29^ and the non-volatile switching devices can retain information for a long time and can be classified as write-once-read-many-times (WORM) memory or rewritable memory devices. Particularly, for the memristive behavior, most of these devices have high OFF/ON resistance ratio and good retention characteristic; therefore, the resistance switching memory devices have significant potential for applications in future non-volatile RRAM memory cells.

In this study, we report a resistive switching memory device based on natural keratin extracted from hair. At first, we fabricated the Ag/keratin/ITO sandwiched structure and performed a typical current–voltage (*I*–*V*) test under ambient conditions. The as-fabricated memory device showed stable unipolar resistive switching behavior and good retention characteristic over 150 consecutive cycles. Moreover, to test the electrode dependence of keratin, FTO and Ti were used as bottom electrodes; moreover, the keratin-based device exhibited stable memristor performance when FTO and Ti were used as bottom electrodes. Therefore, the keratin-based device shows multiple adaptability and flexibility, and it provide a variety of choice for future diversified applications. Finally, Ag and graphite NPs were doped into the keratin intermediate layer to investigate the influence of conductive substances on the memristive performance of this device. Our result shows that the doping of Ag and graphite can increase the conductivity of the dielectric layer and reduce the switching ratio. This indicates flexibility and good adaptability of the memory devices based on keratin, which can be used for information memory; moreover, this demonstrates that the addition of conductive NPs can inhibit the memristive performance of the device. We believe this study will be helpful for the development of future bio-electronic devices and green functional devices.

## 2. Experiment

Keratin powder was extracted from hair using a similar method reported in some previous studies.^30,31^ At first, we cut the obtained hair into small pieces and cleaned them by soaking in a detergent and rinsing with distilled water. Then, the hair was dissolved in a SDS (sodium dodecyl sulfate, 15 g L^−1^), NaOH (10 g L^−1^), and Na_2_SO_3_ (50 g L^−1^) mixed solution. In this solution, the role of NaOH is to destroy the cortical layer of the hair, that of Na_2_SO_3_ is to reduce the disulfide bonds, and SDS can avoid the oxidation of the sulfhydryl group because the reduced sulfhydryl group can have good activity and can be easily oxidized. After mixing the hair in the solution, we first let the mixture of the hair and solution stand for 30 min at room temperature and then maintained the temperature at 80 °C for 3 hours to let the reaction complete. After this, we filtered the hair lysis solution followed by centrifugation (10 000 rpm), and dialysis of the resulting supernatant was carried out in distilled water using a dialysis membrane for 48 hours. Finally, the solution was placed in a dry box fixed at 50 °C for evaporation, and thus, we obtained keratin powder after grinding.

**Fig. 1:**
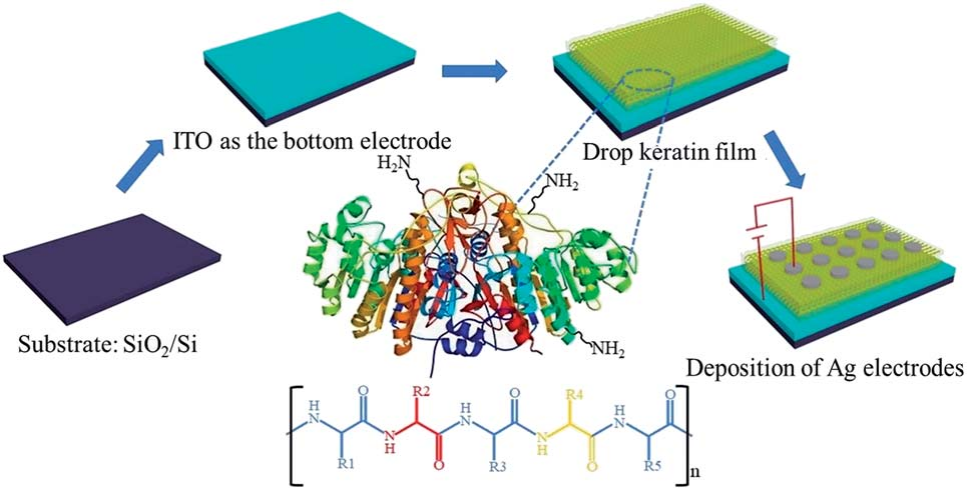
The schematic of the preparation of the Ag/keratin/ITO memory device and the chemical structure of keratin.

The memory device was fabricated on an ITO-coated Si/SiO_2_ substrate. A 185 nm-thick ITO layer acted as the bottom electrode. The substrate was first cleaned in distilled water with ultrasonication for 10 min and evaporated in a drying box; then, the aqueous solution of keratin was spin-coated onto the optically transparent ITO-coated glass substrate at room temperature. The aqueous solution of keratin was prepared by mixing keratin powder with the KClO_4_ electrolyte. After the keratin film was deposited, the sample was dried at 40 °C in a vacuum environment for 24 hours. Finally, the Ag top electrode with the area of ∼1.0 mm^2^ was deposited using thermal deposition at the pressure of ∼10^−4^ Pa. Thus, we obtained a device with the Ag/keratin/ITO sandwiched structure (Fig. 1).

The electrical characteristics of the as-fabricated devices were tested by an electrochemical workstation under normal circumstances using a cyclic scanning model, and the scan rate was 1.0 V s^−1^. During the electrical measurement of the memory device, the direct current (DC) bias voltage was applied to the Ag top electrode, and the ITO bottom electrode was grounded.

## 3. Result and discussion

**Fig. 2:**
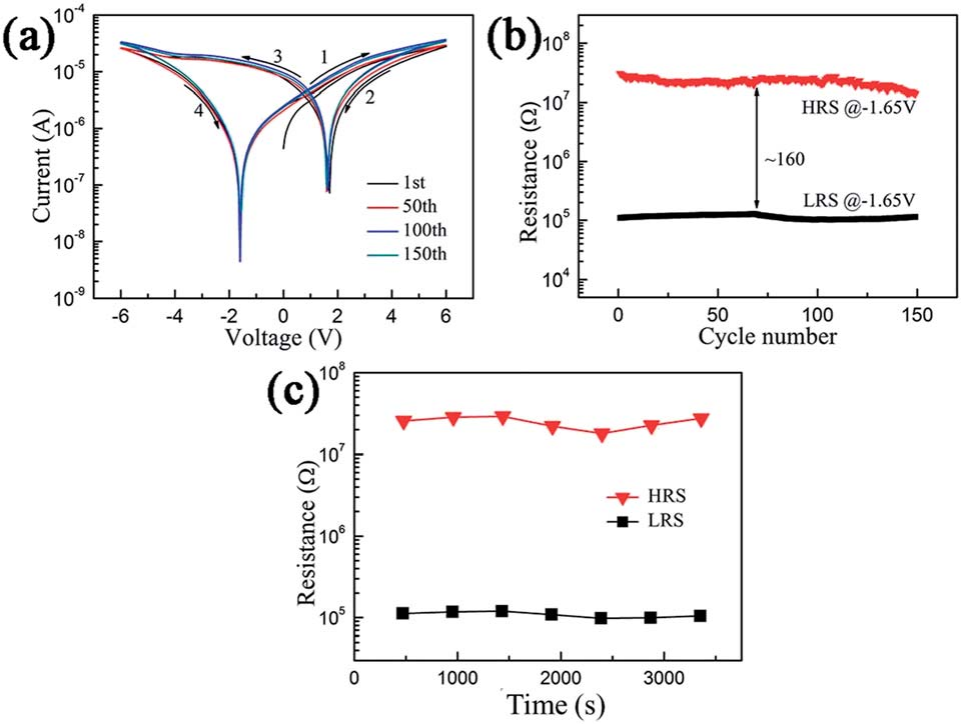
The resistive switching characteristics of the Ag/keratin/ITO memory device. (a) Typical *I*–*V* curves with different laps under single logarithmic coordinates. (b) Distribution of the HRS and LRS over 150 consecutive cycles. (c) Retention characteristics of both resistance states.

The hysteretic behavior of the *I*–*V* curves under cyclic voltage scanning is the most important intrinsic characteristic of memristive effect, showing an obvious nonlinear behavior. Thus, we can observe a high resistance state (HRS) and a low resistance state (LRS) at a specific applied voltage, and the fast conversion between the HRS and the LRS enables the memristor to implement computerized binary for information memory. Therefore, the electrical characteristic of the as-prepared memristor is of great significance. The electrical properties of the memory devices with the Ag/keratin/ITO structure were demonstrated in the direct current (DC) sweeping mode. During electrical measurement, the DC bias voltage was applied to the top electrode Ag, and the bottom electrode ITO was grounded. The as-fabricated devices exhibited a typical unipolar resistive switching effect at the compliance current of 1.0 mA. An *I*–*V* curve on a semi-log scale of the memory device is plotted in Fig. 2a. It is obvious that the memory device with the Ag/keratin/ITO structure presents a stable resistive switching memory window under 0 → 6.0 V → 0 → −6.0 V → 0 cycle voltage scan. We can observe that no obvious set or reset process is shown in the *I*–*V* curves, and it can be seen that the minimum value of current does not appear at 0 V; this can be because of ion aggregation at the interface between the Ag electrode and the keratin layer. During the continuous scanning process, the device can be constantly switched between the HRS and the LRS; this provides two different logic states of the written and erased data for information memory.

To reveal the memory performance of the as-prepared memristor device, we investigated the HRS/LRS resistance ratio and retention characteristics. Fig. 2b shows the distribution of the HRS and LRS over 150 consecutive cycles at the voltage of −1.65 V. Fig. 2c exhibits the retention time characteristic of the device for both resistance states. As shown in the figure, the HRS and LRS are roughly stable during 150 cycles, the OFF/ON resistance ratio is about 160, and the retention time for all resistance states can reach 3000 s. Because of the large resistive switching ratio and good retention characteristic, the proposed device has the potential to be applied in biomaterial-based nonvolatile memory devices.

The charge transport mechanism of dielectric films can be an important basis for us to analyze the switching window of the memory device. To date, the conductive mechanism of memristor devices was mainly focused on metal filament formation, redox reactions and trapping/detrapping processes.^32,33^ Previously, for protein-based memristor devices, the trapping/detrapping process of the defect state was the main conduction mechanism.^11,14,34^ Because proteins have a certain spatial structure, it will cause a large number of defects in the dielectric layer prepared by proteins. Therefore, in our device, we can confirm the existence of defective states in the keratin intermediate layer. On the other hand, because the Ag atoms can be easily ionized and converted into Ag ions, the formation of Ag conductive filaments is highly possible; thus, in our device, Ag conductive filaments can also be formed.

**Fig. 3:**
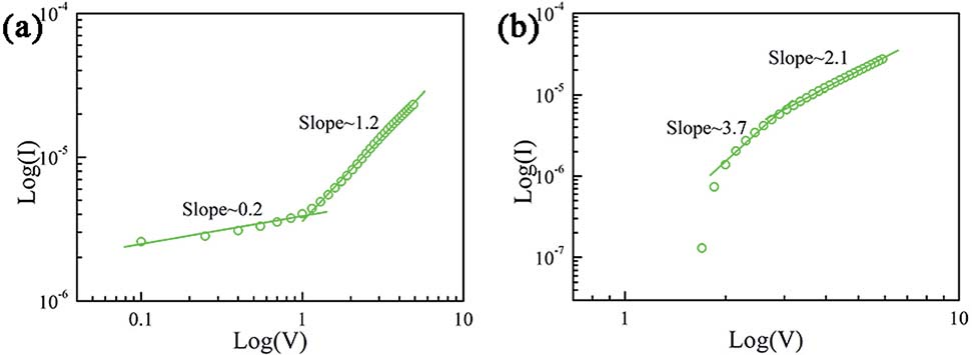
The *I*–*V* curve for the Ag/keratin/ITO memory device on a double logarithmic scale. (a) LRS at positive bias. (b) HRS at positive bias.

To explore the mechanism, at first, we performed linear fitting in the positive bias region in a double logarithmic scale, as shown in Fig. 3. Fig. 3a shows the linear fitting of the LRS part in double logarithmic coordinates. The curve fitting can be divided into two distinct parts: the slope of linear fitting is ∼0.2 in the 0–1.2 V range (low bias) and ∼1.2 in the 1.2–6.0 V range (high bias). In the low bias region, the electrons injected from the electrode are first captured by the defect center; moreover, a conduction path is formed after the defect is filled, which follows hopping conduction. In the high bias region, the fitting slope is obey the ohmic conduction. This shows that metal conduction filaments can be formed in our device. The slope is not equal to one; this is caused by the drifting motion of the electrons released by the defect at high bias. Fig. 3b shows the linear fitting of the HRS part in a double logarithmic scale, and this linear fitting can also be divided into two parts: the slope of the linear fit is ∼2.1 in the 1.2–6.0 V range and ∼3.7 in the 1.1–1.2 V range. This indicates space-charge limited conduction (SCLC) behavior, and the fitting slope is higher at low biases of the HRS part; this may be attributed to the built-in electric field, which makes the current decrease rapidly. Based on the abovementioned linear fitting analysis and the defect state of the keratin layer, when our fabricated device is in the low resistance state, hopping conduction caused by the defect mainly works in the low bias region, and the conductive filaments act primarily in the high bias regions because a large voltage will cause the trapped electrons to pass through a potential well; on the other hand, the formation of metal conductive filaments needs a long period of time. In the HRS range, the curve follows an SCLC behavior. In addition, the thermal effect and ion aggregation at the interface affect the switching process.

**Fig. 4:**
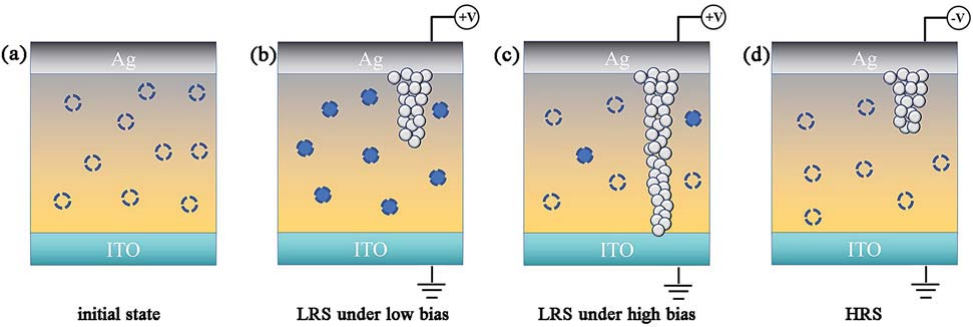
The switching mechanism of the Ag/keratin/ITO memory device. The hollow dotted round circle represents the defect center. The solid dotted round circle represents the defect center occupied by charge carries.

Fig. 4 illustrates the switching mechanism of our fabricated memory device. At the beginning, there are some unfilled defect states disorderly distributed in the keratin dielectric layer, as shown in Fig. 4a. In the low voltage range, when positive voltage is applied to the Ag electrode, the defect state will be rapidly filled with electrons. Subsequently, the electrons injected from the cathode can be transported to the anode through the defect center.^11^ Moreover, the Ag conductive filaments are formed in the dielectric layer, as shown in Fig. 3b. However, when the voltage continues to increase, under a lager electric field, the electrons captured by the defect center would be released, and the released electrons will drift to the anode under an electric field. This can also explain why our fitting slope is not exactly equal to ∼1.0. In addition, the Ag conductive filaments have been formed, and now, the conduction mechanism is mainly dominated by Ag conductive filaments, as shown in Fig. 3c. When the scan voltage is reversed, after the established conductive filaments rupture, the curve follows the SCLC behavior, as shown in Fig. 4d.

**Fig. 5:**
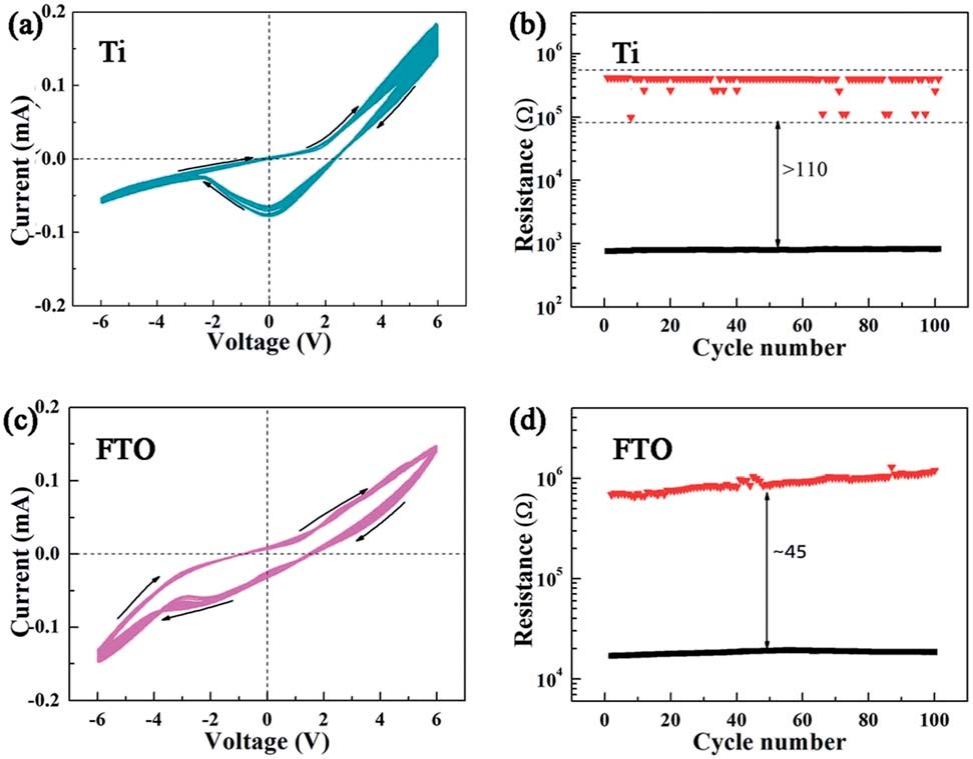
(a and b) The *I*–*V* curve for a keratin-based memristor and the distribution of the HRS and LRS over 100 consecutive cycles with Ti as the bottom electrode. (c and d) The *I*–*V* curve for a keratin-based memristor and the distribution of the HRS and LRS over 100 consecutive cycles with FTO as the bottom electrode.

In addition, to prepare a flexible keratin-based memory device, a Ti sheet was used as a substrate. Moreover, we fabricated Ag/keratin/Ti and Ag/keratin/FTO structure devices. The I–V curves are shown in Fig. 5a for Ti and Fig. 5c for FTO. It can be observed that a negative current appears at a positive bias, and its formation is mainly related to the ions in the keratin layer. Moreover, Fig. 5b and d show the distribution of the HRS and LRS with cycle number. We can observe that the HRS/LRS resistance ratio is approximately 110 for Ti as the bottom electrode, whereas it is ∼45 for FTO as the bottom electrode. Although the distribution of the HRS and LRS reflects slight instability, keratin shows a good switching memory window. Based on the abovementioned description, it can be concluded that keratin exhibits stable memristor properties on different bottom electrodes; this proves that keratin-based memristor devices possess adaptability and flexibility and can provide more technical solutions for future applications.

It is usually necessary to change certain variables to study the response of memristor devices to the external environment. The most frequently changed variables are temperature, chemical reagent modification, compliance current, and set or reset voltage.^33–35^*Via* this, a controllable memristor behavior can be obtained, which is of great significance for future adjustable memristor devices. Previously, some researchers have reported and used gold, silver, copper, and aluminum metal nanoparticles doped into an intermediate medium layer to modulate the memory performance of devices;^8,36,37^ however, the difference in the device fabrication process and the uncertainty of the microscopic switching mechanism often leads to different conclusions.

**Fig. 6:**
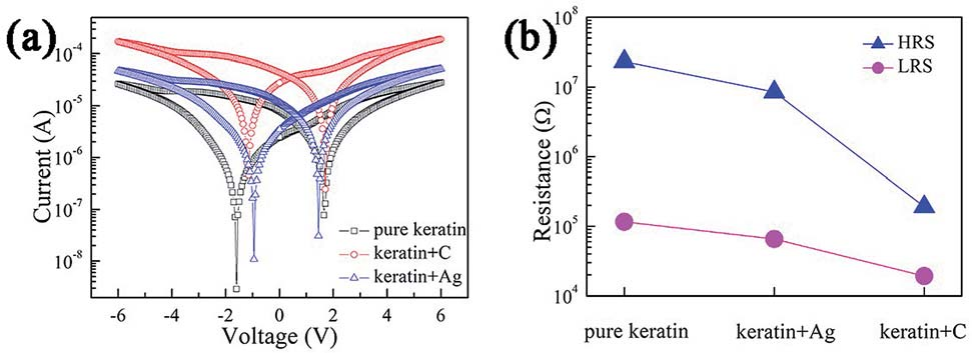
(a) *I*–*V* curves for the doped device and undoped devices. Graphite is denoted by the abbreviation “C.” (b) Resistance for the HRS and the LRS, represented by the blue curve and magenta curve, respectively.

In this study, Ag NPs and graphite NPs with good conduction property were chosen to be doped into the keratin layer. The *I*–*V* curves for Ag and graphite NP-doped keratin films and pure keratin film are shown in Fig. 6a. These curves are derived from the results of the second cycle scan. As shown in Fig. 6, the current of the *I*–*V* curve increases after the introduction of Ag and graphite NPs, and the capacitance effect shows a weakening trend. Based on the abovementioned data, it can be seen that by doping conductive particles into the keratin layer, the electrical conductivity of the memristor devices can be effectively enhanced, whereas the capacitance effect can be reduced. This may be because conductive particles contribute to the formation of conductive filaments and electron transport.^38–40^ We also studied the resistance of the HRS and LRS of the doped device, as shown in Fig. 6b. After doping Ag NPs and graphite NPs, the HRS and LRS are reduced, and the OFF/ON resistance ratio is decreased: it is ∼120 for the Ag NP-doped keratin film and ∼10 for the graphite NP-doped keratin film. Compared with the previously reported pure keratin film-based device (the OFF/ON resistance ratio is ∼180), the device based on the doping of Ag/graphite NPs exhibits decreased memory performance.

## 4. Conclusions

In this study, we report a resistive switching memory device based on keratin. The as-fabricated device shows excellent resistive switching memory behavior and stable OFF/ON resistance ratio over 150 consecutive cycles, and the retention time can reach 3000 s. Moreover, the keratin-based memristor device exhibits stable switching performance with Ti and FTO as the bottom electrodes. The switching mechanism is dominated by hopping conduction at a low bias (0.0–1.2 V) and conductive filaments at a high bias (1.2–6.0 V). Furthermore, we revealed the inhibition of switching performance by doping Ag/graphite NPs. Thus, as a renewable natural resource, keratin can be applied in memristor switching devices to alleviate the problem of overcapacity; moreover, this will guide researchers to make rational utilization of the discarded resources. We believe that this green and pollution-free memory device could be a big step forward to the development of green electronic devices and a good example for better utilization of other waste resources.

## Conflicts of interest

There are no conflicts to declare.

## Supplementary Material
